# Efficacy and Effect of Inhaled Adenosine Treatment in Hospitalized COVID-19 Patients

**DOI:** 10.3389/fimmu.2021.613070

**Published:** 2021-03-18

**Authors:** Massimo Caracciolo, Pierpaolo Correale, Carmelo Mangano, Giuseppe Foti, Carmela Falcone, Sebastiano Macheda, Maria Cuzzola, Marco Conte, Antonella Consuelo Falzea, Eleonora Iuliano, Antonella Morabito, Michele Caraglia, Nicola Polimeni, Anna Ferrarelli, Demetrio Labate, Marco Tescione, Laura Di Renzo, Gaetano Chiricolo, Lorenzo Romano, Antonino De Lorenzo

**Affiliations:** ^1^Unit of Post-Surgery Intensive Therapy (USDO), Grand Metropolitan Hospital, Reggio Calabria, Italy; ^2^Medical Oncology Unit, Grand Metropolitan Hospital, Reggio Calabria, Italy; ^3^Unit of Infectious Disease, Grand Metropolitan Hospital, Reggio Calabria, Italy; ^4^Unit of Radiology, Grand Metropolitan Hospital, Reggio Calabria, Italy; ^5^Unit of Intensive Care Medicine and Anaesthesia, Grand Metropolitan Hospital, Reggio Calabria, Italy; ^6^Microbiology Unit, Grand Metropolitan Hospital, Reggio Calabria, Italy; ^7^Unit of Pharmacy, Grand Metropolitan Hospital, Reggio Calabria, Italy; ^8^Department of Precision Medicine, University of Campania “L. Vanvitelli”, Naples, Italy; ^9^Biogem Scarl, Institute of Genetic Research, Laboratory of Precision and Molecular Oncology, Ariano Irpino, Italy; ^10^Section of Clinical Nutrition and Nutrigenomics, Department of Biomedicine and Prevention, University of Rome Tor Vergata, Rome, Italy; ^11^Department of Biomedicine and Prevention, University of Rome Tor Vergata, Rome, Italy; ^12^PhD School of Applied Medical, Surgical Sciences, University of Rome Tor Vergata, Rome, Italy

**Keywords:** adenosine, COVID-19, SARS-CoV-2, length of stay, inflammation

## Abstract

Lack of specific antiviral treatment for COVID-19 has resulted in long hospitalizations and high mortality rate. By harnessing the regulatory effects of adenosine on inflammatory mediators, we have instituted a new therapeutic treatment with inhaled adenosine in COVID-19 patients, with the aim of reducing inflammation, the onset of cytokine storm, and therefore to improve prognosis. The use of inhaled adenosine in COVID19 patients has allowed reduction of length of stay, on average 6 days. This result is strengthened by the decrease in SARS-CoV-2 positive days. In treated patients compared to control, a clear improvement in PaO_2_/FiO_2_ was observed together with a reduction in inflammation parameters, such as the decrease of CRP level. Furthermore, the efficacy of inhaled exogenous adenosine led to an improvement of the prognosis indices, NLR and PLR. The treatment seems to be safe and modulates the immune system, allowing an effective response against the viral infection progression, reducing length of stay and inflammation parameters.

## Introduction

A new pandemic, Coronavirus disease 2019 (COVID-19), caused by severe acute respiratory syndrome following infection with the Severe acute respiratory syndrome coronavirus 2 (SARS-CoV-2) virus, hit the world starting in December 2019 (1, 2).

COVID-19 has shown an unexpected long incubation that has allowed the virus to be carried. Additionally, SARS-CoV-2 has shown the ability to trigger severe and inappropriate immune-inflammatory response in about 17–20% of infected individuals. This in turn may lead to highly lethal conditions including systemic micro-vascular damage with multiorgan failure and the dreaded acute respiratory distress syndrome (ARDS) which presents a 50% lethality rate (3).

After the outbreak of the SARS-CoV-2, extensive researches were conducted to find common associations with SARS-CoV. In the viral genome, the 5 terminal portion encodes non-structural proteins involved in some viral processes, and the remaining part encodes structural and accessory proteins, S (Spike), E (Envelope), M (Membrane) and N (Nucleocapsid) (4). Structural trimeric protein S, composed of domains associated with different functions, such as virus entry and host tropism, determines the corona layer and the name of the virus. Through the ectodomain, composed of the S1 and S2 subunits, respectively receptor-binding and membrane-fusion domains, the virus interacts with specific receptors of the host target tissues (5).

SARS-CoV-2 hits different target organs, and mainly affects the lungs, small intestine and arterial smooth muscle cells, due to the increased expression of angiotensin converting enzyme 2 (ACE) (6), a zinc-containing metalloenzyme, which promotes viral infection (7). Furthermore, a possible bond between S1 viral subunit and host's Neuropillin-1 has recently been studied (8).

Therefore, within the infected population, COVID-19 can be asymptomatic or it can present multiple grades of severity and a wide spectrum of organ specific clinical manifestations as mildly asymptomatic, with fever, cough, fatigue, and intestinal symptoms; severely symptomatic, with progressive respiratory failure, pneumonia, ARDS, up to multiple organ failure and death (1, 9).

Recently, other clinical features have been described, including coagulopathies (due to reduced levels of platelets and fibrinogen), higher levels of lactate dehydrogenase (LDH), aspartate aminotransferase (AST) and alanine aminotransferase (ALT) whose role within the intraindividual clinical evolution of COVID-19 and its severity still need to be clarified (10).

The comorbidities, such as obesity, hypertension, chronic lung disease, diabetes mellitus, kidney disease, and cardiovascular disease, complicate the clinical course of COVID-19 (11).

Patients with obesity co-morbidities are more exposed to an evolution in ARDS from COVID-19, with fraction of inspired oxygen (FiO_2_) lower than 60%, need for mechanical ventilation and intensive care unit (ICU) hospitalization (12).

Dramatically, over 50% of COVID-19 patients needed respiratory support due to an excessive pro-inflammatory response, with a massive release of interleukins (IL)-1β, IL-6 and Tumor Necrosis Factor (TNF)-α, chemokines (13) and anti-inflammatory cytokines (1).

When there is a hyper-inflammatory state with maladaptive release of cytokines, the “cytokine storm” occurs, affecting mainly the lungs. The name is borrowed for its homologies with a similar clinical condition described in cancer patients under immunological treatment with cytokines (high does IL-2 and IL-12), costimulatory molecules (anti-CD28) and immune-checkpoint inhibitors (14).

This phenomenon had previously been observed also with SARS-CoV, involving IFN-γ and IL-18 (15). The overproduction of IL-6 can increase the risk of cardiovascular diseases, with high levels of hepatic C-reactive protein (CRP) (16).

Several reports suggest that the dysfunctional inflammatory process and the uncoordinated release of cytokines can severely compromise the innate or the humoral immunity. Therefore, the immune-surveillance system may not mount an efficient response to SARS-Cov-2 able to clear the infection, and to maintain an immunological lasting memory (17).

Furthermore, the onset of the cytokine storm compromises the antiviral response with a malfunctioning innate immune response. Natural killer cells, monocytes and macrophages as the first line of defense, predispose to the development of severe pneumonia, due to the alteration of Interferon levels (18).

The altered immune response, together with elevated levels of proinflammatory molecules as IL-1B, IFN-γ, IL-12, IFN-γ, and chemokines have already been observed in infections from SARS-CoV and Middle East respiratory syndrome-related coronavirus (MERS-CoV) (1, 19). Similarly, increased monocyte chemoattractant protein-1 (MCP-1) was observed for all three viruses. This leads to activation and recall of monocytes, modulation of the Th1 mediated response and increase in the expression of inflammatory related Th2 markers (observed only in SarS-CoV2) (1).

Many therapies already in use against COVID-19, have been applied as potential treatments and can be grouped into the two categories of antiviral and immune-based therapies (20). Against SARS-CoV-2 infection, hydroxychloroquine (HCQ) and chloroquine, approved in emergency by the FDA last March, have been tested in several clinical studies (21) but the study reported by Self et al. in Journal of American Medical Association on November 9, 2020 did not show any significant improvement in clinical status in a well-designed placebo-controlled study (22).

Other potential treatments have been tested, many of them under study. These include two protease inhibitors, lopinavir and the more recent darunavir, both already in use against HIV-1 (23).

The combination lopinavir/ritonavir, designed for HIV, inhibits the aspartic protease, while the SARS-CoV-2 possesses a protease 3CLpro acting as cysteine protease. This undermines the effectiveness of the treatment (23, 24) but could lay the groundwork for the design of more active molecules (24). The effects of the combined use of lopinavir/ritonavir with other treatments was controversial and of less relevance as observed in the randomized studies (25).

Promising initial studies to have shown modest efficacy. There was a reduction in recovery time, from a median of 15 days among placebo recipients to 11 days among those receiving remdesivir, and it may have prevented progression to a more severe degree of respiratory disease (26, 27).

Immune-based therapies include monoclonal antibodies (mAb), such as Tocilizumab (TCZ), a humanized mAb acting on the IL-6 receptor (IL-6R), already used for autoimmune diseases (28). In COVID-19, the high release of IL-6 by T lymphocytes contributes to the cytokine storm. Consequently, TCZ competing for the IL-6R minimizes the inflammatory response to prevent possible respiratory insufficiency and ARDS, due to the involvement of the alveolar respiratory membrane (29).

Additional therapies such as anti-IL1β, Canakinumab and Janus kinase inhibitors (JAK) −1/2 (Baricitinib and Ruxolitinib) have been tried out. However, to date, there is insufficient data to support their use (30, 31).

Due to paucity of approved therapies and a well-recognized anti-inflammatory action of adenosine, its application in COVID-19 has been hypothesized. The marked ability to inhibit inflammation is known from ischemia and reperfusion models. Therefore, adenosine has the potential act in acute lung injury as a local anti-inflammatory, also stemming the cytokine storm (32–34).

Adenosine exerts its action through the interaction with four subtypes of adenosine receptors (AR) (35). AR signaling occurs through the pathways of adenylate cyclase, phospholipase C, Ca2^+^ and mitogen-activated protein kinase (36, 37). Among the various effects of adenosine, the anti-inflammatory action is mainly mediated by the AR 2A subtype. This receptor is primarily localized on immune cells, such as lymphocytes, monocytes, macrophages, and dendritic cells. AR 2A activation attenuates inflammatory processes and reperfusion damage inhibiting inflammatory cytokines and activated immune cells (38). Furthermore, in the lung tissue, it improves the functions of the capillary alveolar barrier, restoring physiological gas exchanges. Finally, adenosine-AR 2A coupling is hypothesized to reduce T-cell receptor-mediated (TCR) production of IFN-γ, modulating the activation of T cells and secondary macrophages (39). It is hypothesized that in acute inflammation it may improve the inflammatory and immune response of the host against COVID-19 (40).

The aim of this case-control study is to evaluate the potential improvement in outcome, clinical characteristics and prognosis of hospitalized COVID-19 patients after treatment with aerosolized adenosine at the dosage of 9 mg every 12 h in the first 24 h and subsequently, every 24 h for 5 days.

The primary endpoints are reduction of length of stay (LoS) and improvement of prognosis. The secondary endpoints are the reduction of inflammation severity, and timing of lung lesions by chest Computer Tomography (CT).

## Materials and Methods

### Study Assessments and Procedures

The study population was represented by hospitalized native COVID-19 patients meeting eligibility criteria.

This single-center case-control study involved off-label treatment with adenosine, administered to Sars-CoV2 positive patients, who arrived at the Emergency Department of the “Bianchi Melacrino Morelli” Grande Ospedale Metropolitano (GOM) of Reggio Calabria, Italy, between March 19 and April 13, 2020.

After Sars-CoV2 positivity confirmation, patients were transferred to the Infectious Diseases Unit, underwent chest CT, drug therapy with low molecular weight heparin, azithromycin, HCQ, and lopinavir/ritonavir.

At baseline, after interrupting Lopinavir/ritonavir administration, biochemical-clinical analyses were performed to all eligible patients and off-label treatment was started in the treated group (TG), represented by the patients who accepted compassionate treatment. The control group (CG) included eligible patients, with characteristics homogenous to TG, who did not accept the off-label compassionate use of Adenosine.

The following additional information was collected (41): myocardial infarction (MI); atrial fibrillation; type 2 diabetes mellitus; chronic liver disease; chronic obstructive pulmonary disease (COPD); chronic kidney failure (CKF); stroke; cancer in the last 5 years.

At 10 days from baseline, patients underwent follow-up of blood chemistry and clinical parameters.

On the 21st day after admission to the Infectious Diseases Unit, all patients underwent a chest CT-scan.

The off-label treatment was administered after obtaining informed consent and approval for each patient from the Hospital Safety Team.

All patients' history and clinical status data were collected at baseline and follow up. All chest CT scans, SarS-CoV2 research, and blood chemistry analyses were performed at the GOM.

**Figure 1:**
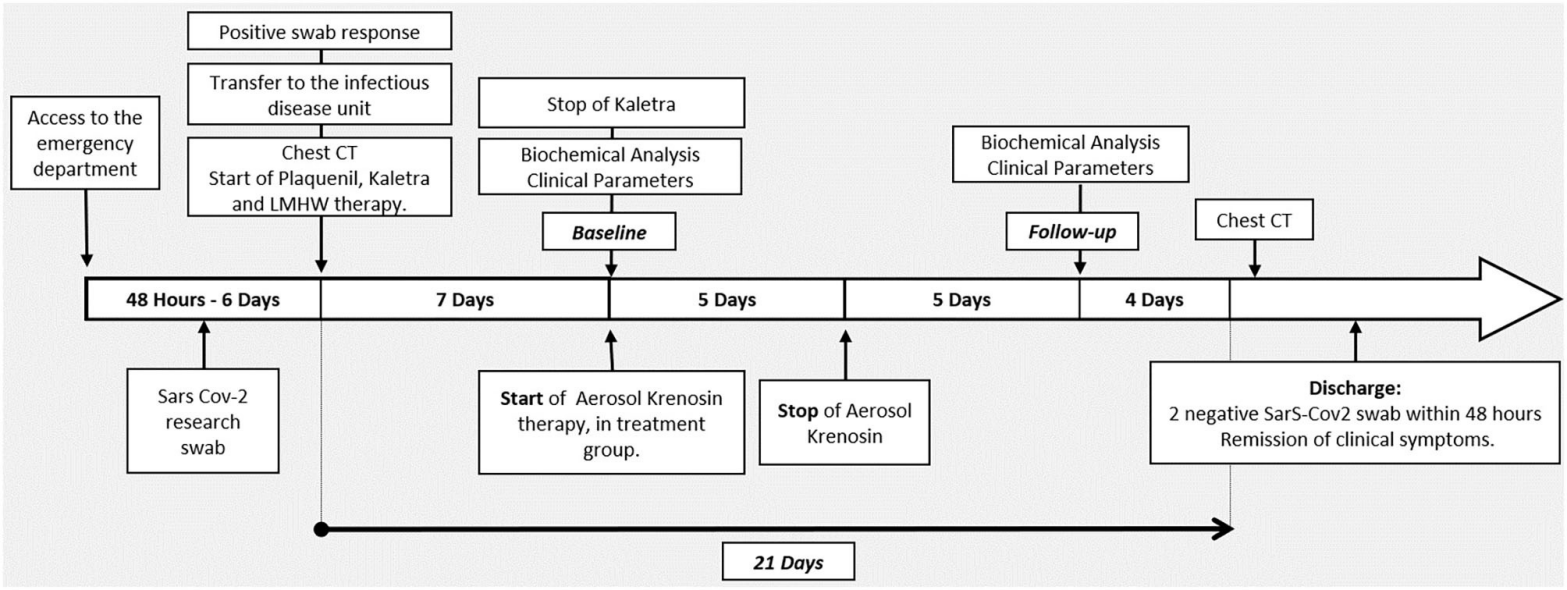
Timeline of progress from admission to discharge.

Days of SarS-CoV2 positivity are evaluated from the first positive test to the second negative test in 48 h. In addition, hospitalization days were examined from transfer to the Infectious Diseases Unit until discharge. The discharge was allowed with two negative SarS-CoV2 tests within 48 h and remission of clinical symptoms. Timeline of progress from admission to discharge is showed in Figure 1. The study was approved by the Ethics Committee of Southern Calabria (April 30, 2020). The privacy and sensitive data of patients have been protected and the database can be consulted on the IT System of GOM, after appropriate authorization (direzionesanitaria@ospedalerc.it), according to the GDPR UE 2016/679.

Inclusion criteria:

- Biologically confirmed by SARS-CoV-2 PCR test.
- COVID-19 diagnosis clinically and radiologically confirmed by chest CT scan.

Exclusion criteria:

- Patients with a history of neutropenia.
- Patients with acquired immunodeficiency.
- Patients with cancer history in the last 5 years.
- Patients who underwent transplants.
- Patients who received previous immunosuppressive therapies or corticosteroids.
- Women who are pregnant or breastfeeding.

### Withdrawal Criteria

Participants were free to withdraw from participation in the study at any time upon request or at the request of their legally acceptable representative.

An investigator may discontinue or withdraw a participant from the study for the following reasons:

- Pregnancy
- Non-compliance to study intervention
- Occurrence of any clinical adverse event, laboratory abnormality, or medical condition compromising continued participation in the study and not in the best interest of the participant.
- Severe side effects clearly related to the study device.
- Disease progression requiring other treatments.
- If the participant meets an exclusion criterion (either newly developed or not previously recognized) that precludes further study participation
- Participant unable to receive study intervention for > 2 days/week.
- Subject non-compliant to investigational procedures.
- Subject non-compliant to visits.

### Therapy

All patients received standard therapeutic protocol including low molecular weight heparin 4,000 units two times a day, azithromycin 1 tablet a day when needed, HCQ (Plaquenil®) 200 mg, 2 tablets two times a day, lopinavir/ritonavir (Kaletra®) 100/25 mg, 2 tablets two times a day for 7 days (42).

Respiratory support was used when needed, according to current guidelines.

The off-label treatment involved the use of inhaled adenosine (Krenosin®), 9 mg every 12 h for the first 24 h and subsequently, every 24 h for 4 days (43, 44). Nebulized adenosine was delivered by an Aerogen USB® controller connected to a high flow 60 L for 5 min device, with 21% FiO_2_. The safe posology has been established from preclinical studies (45, 46).

The administration of aerosolized adenosine has a dose-limiting efficacy >10 mg. Its use did not show adverse effects in non-asthmatic subjects (47, 48).

Adverse effects were monitored throughout the study and patients underwent continuous monitoring of cardiocirculatory and respiratory function.

### Sars-CoV2 Swab Test

SARS-CoV-2 was detected by RNA sequencing assay (Seegene Allplex™ 2019-CoV Assay, catalog number #RP10243X 100 rxn), targeting SARS-CoV-2 RdRp, E and N genes. Data were analyzed by CFX96 Manager software and Seegene viewer software. The responses were “2019-nCoV detected,” “negative,” or “invalid” (Allplex 2019-nCoV assay IFU) (49).

### Anthropometry

The Body Mass Index (BMI) was calculated by weight and height measured, according to Romano et al. (50) or referred when unable to carry out the assessment.

### Biological Specimen Collection and Laboratory Evaluations

The following blood analysis were performed at each evaluation time: blood gas analysis parameters, as arterial oxygen partial pressure (PaO_2_) and arterial carbon dioxide partial pressure (PaCO_2_); fractional inspired oxygen (FiO_2_) in patients with respiratory support; arterial oxygen partial pressure and fractional inspired oxygen ratio (PaO_2_/FiO_2_) (51); platelets (10^3^/μL); white blood cells (WBC) (10^3^/μL); red blood cells (RBC) (10^6^/μL); hemoglobin (g/dL); neutrophils (10^3^/μL); lymphocytes (10^3^/μL); monocyte (10^9^/L); fibrinogen (mg/dL); D-dimer (ng/mL); CRP(mg/L); glycemia (mg/dL); albumin (g/dL); AST (U/L); HALT (U/L); gamma-glutamyl transpeptidase (GGT); indirect bilirubin (mg/dL); direct bilirubin (mg/dL); amylase (U/L); lipase (U/L); potassium (mEq/L); sodium (mEq/L). According to Qin, neutrophils to lymphocytes (NLR) and platelet to lymphocyte (PLR) were calculated (52).

### Chest CT and Findings

CT examination was performed with high-resolution acquisitions for the study of lung interstitium, with CT GE Medical System Optima CT 660, followed by multi-parametric reconstructions according to coronal and sagittal and 3D plans.

Two radiologists independently and blindly examined the Chest CT scans and reported the radiographic findings, according to Chung (53).

### Statistical Analysis

All statistical analyses were conducted with SPSS 23 software (version 23.0, IBM, Armonk, NY, USA). Data collected before statistical evaluations were analyzed for the presence of outliners and for normally distribution with the Shapiro–Wilk test. The data presented are expressed as mean, standard deviation, percentage in contingency tables and as Δ%, to evaluate differences between the times. Before, the differences between CG and TG patients were assessed by Independent samples *t*-test and Mann Whitney test. Subsequently, the two-tailed Student's paired *t*-test or Wilcoxon rank test were used to assess the presence of differences in the variables examined between baseline and follow-up. Conclusively, for each study variable, to compare the trend over time, Δ% were calculated equal to the percentage variation of each parameter calculated as an absolute margin of variation from the baseline value. The differences in Δ% between baseline and follow-up among groups were assessed with the ANOVA one-way test. The presence of difference in contingency tables were analyzed with the Chi square test. Statistical significance was set to a value of *p* < 0.05. All *p*-values shown are two-tailed.

## Results

**Figure 2:**
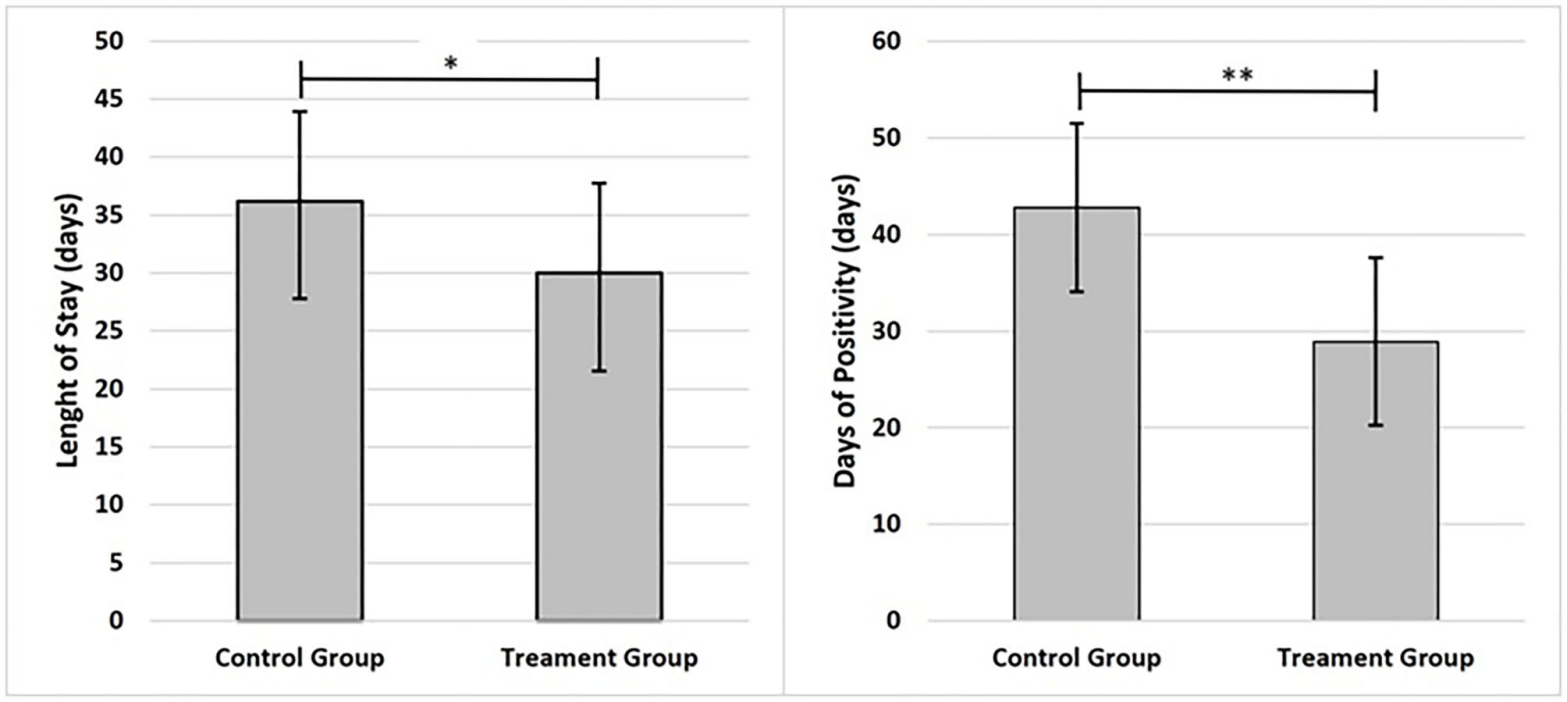
Graphs present main results; data are shown as mean and standard deviation ±1. Statistical significance was attributed as **p < 0.05; **p < 0.01.

Of the 30 Caucasian patients enrolled for prospective analytical case-control study, 6 subjects were excluded from the study for the following reasons: 2 subjects did not meet inclusion criteria (transfer in intensive care unit), 1 subject died before starting treatment; 3 subjects were excluded for incomplete data. Finally, 24 patients were included in the study. The age of subjects was 56.86 ± 15.65 years, 37.50% females and 62.50% males. The eligible patients were allocated into two groups: 12 patients in the CG (41.67% females and 58.33% males) and 12 patients in the treatment group (TG) (33.33% females and 66.67% males). As reported in Figure 2, it was observed that the days of hospitalization and test positivity were statically decreased in TG compared to CG (respectively *p* = 0.032; *p* = 0.002). As reported in Table 1, No statistical differences between groups were observed in age, BMI, days of HDCL and AZT treatment. At baseline, only PLR was statistically increased in TG compared to CG (*p* = 0.004). Lymphocytes and potassium were significantly reduced in TG compared to CG (respectively *p* = 0.021; *p* = 0.022). No other statistical differences between groups were detected. As reported in Table 2, at baseline no statistical differences were observed in the frequency of airway support, hypertension, diabetes mellitus and dementia. Furthermore, none of the enrolled patients suffered from COPD, CKF, MI and cancer in the past 5 years. As reported in Table 3, between baseline and follow-up, it was observed that NLR, RBC, hemoglobin and neutrophils were statically reduced in CG (respectively *p* = 0.028; *p* = 0.032; *p* = 0.032; *p* = 0.038), the PaO_2_/FiO_2_, platelets and lipase concentration were statically increased in CG (*p* = 0.042; *p* = 0.038; *p* = 0.037). Between baseline and follow-up, NLR, PLR, FiO_2_, CRP, amylase, lipase and D-dimer concentration were statistically decreased in TG (respectively *p* = 0.008; *p* = 0.003; *p* = 0.042; *p* = 0.005 *p* = 0.025; *p* = 0.042; *p* = 0.020); PaO_2_, PaO_2_/FiO_2_ and lymphocytes were significantly increased in TG (respectively *p* = 0.045; *p* = 0.014; *p* = 0.013). Moreover, PLR and CRP showed a greater significant Δ% reduction in TG (respectively *p* = 0.017; *p* = 0.046). It was observed that Δ% PaO_2_/FiO_2_ was significantly raised in TG (*p* = 0.046) and Δ% platelets in CG (*p* = 0.013) (Table 3). In Table 4, qualitative changes in CT findings are reported. It was observed that ground-glass opacities or consolidation and pleural effusion frequencies were overall, and in each group, statically reduced (respectively *p* = 0.002; *p* = 0.021; *p* = 0.021 and *p* = 0.007; *p* = 0.021; *p* = 0.048). Ground-glass opacities with consolidation, rounded morphology and pneumopathy >25% frequencies were significantly decreased in overall and TG (respectively *p* = 0.012; *p* = 0.045; *p* = 0.023; *p* = 0.012; *p* = 0.041 and *p* = 0.048). More than two lobes affected, and bilateral lung disease frequencies were statistically increased in CT (*p* = 0.035). Pneumopathy frequencies was observed statically reduced only in overall (*p* = 0.029).

**Table 1:** Differences between control group and treatment group at baseline.

**Parameters**	**Control group**	**Treatment group**	
	**Means ± SD**	**Means ± SD**	***p***
Age (years)	58.80 ± 15.84	54.92 ± 16.90	0.578
Days HDCL	11.50 ± 3.89	10.11 ± 3.30	0.922*
Days AZT	11.50 ± 5.96	11.11 ± 4.26	0.767*
**Baseline**			
BMI (kg/m^2^)	30.08 ± 5.02	31.59 ± 3.74	0.427
NLR	4.32 ± 3.94	7.14 ± 8.86	0.644*
**PLR**	94.03 ± 84.58	305.17 ± 166.07	0.004*
PaO_2_ (mmHg)	86.20 ± 17.12	79.25 ± 12.01	0.351
PaCO_2_ (mmHg)	39.20 ± 4.76	42.08 ± 11.73	0.670*
FiO_2_ (%)	0.31 ± 0.12	0.29 ± 0.12	0.655*
PaO_2_/FiO_2_	304.35 ± 109.35	315.67 ± 123.06	0.916*
RBC (10^3^/μL)	4.85 ± 0.38	4.43 ± 0.73	0.110
Hemoglobin (g/dL)	13.65 ± 1.73	13.23 ± 2.39	0.644
Platelets (10^3^/μL)	147.52 ± 126.17	230.92 ± 122.42	0.132
WBC (10^3^/μL)	8.78 ± 4.41	5.58 ± 2.94	0.055
Neutrophils (10^3^/μL)	6.25 ± 4.57	3.98 ± 3.10	0.183
**Lymphocytes (10**^**3**^**/μL)**	1.74 ± 0.63	1.01 ± 0.72	0.021
C-reactive Protein (mg/L)	59.13 ± 76.79	49.70 ± 47.71	0.927*
Glycemia (mg/dL)	107.3 ± 13.71	99.58 ± 14.11	0.211
Albumin (g/dL)	4.01 ± 0.47	3.82 ± 0.63	0.435
Sodium (mEq/L)	135.8 ± 4.89	134.58 ± 4.29	0.542
**Potassium (mEq/L)**	4.28 ± 0.52	3.80 ± 0.38	0.022
AST (U/L)	34.6 ± 27.35	53.67 ± 43.60	0.284*
ALT (U/L)	42.50 ± 31.11	59.50 ± 56.02	0.767*
GGT (U/L)	43.50 ± 26.69	38.17 ± 29.03	0.468*
Indirect bilirubin (mg/dL)	0.64 ± 0.39	0.91 ± 0.77	0.262*
Direct bilirubin (mg/dL)	0.27 ± 0.09	0.23 ± 0.14	0.552*
Amylase (U/L)	87.2 ± 44.34	76.92 ± 43.84	0.322*
Lipase (U/L)	178.5 ± 193.15	143.42 ± 105.84	1.00
Fibrinogen (mg/dL)	554.00 ± 265.23	459.25 ± 231.85	0.448
D-Dimer (ng/mL)	736.20 ± 564.55	346.67 ± 309.91	0.083

All parameters are presented as mean ± standard deviation, they were compared by Independent samples t-test and (*) Mann Whitney test, statistical significance was attributed as p <0.05.

*SD, standard deviation; BMI, body mass index; HDCL, hydroxychloroquine; AZT, azithromycin; PaO_2_, arterial oxygen partial pressure; PaCO_2_, arterial carbon dioxide partial pressure; FiO_2_, fractional inspired oxygen; PaO_2_/FiO_2_, arterial oxygen partial pressure and fractional inspired oxygen ratio; NLR, neutrophils and lymphocytes ratio; PLR, platelets and lymphocytes ratio; RBC, red blood cells; WBC, white blood cells; AST, aspartate aminotransferase; ALT, alanine aminotransferase; GGT, gamma-glutamyl transpeptidase*.

**Table 2:** Contingency of the respiratory support, Azithromycin (AZT) and comorbidity and needs at baseline.

	**Group**				
		**Control**	**Treatment**	**Tot**	
Airways support	No (%)	27.30	31.75	59.05	
	Yes (%)	18.20	22.75	40.95	χ^2^ = 0.006
	Tot (%)	45.50	54.50	100.00	*p* = 0.640
AZT	No (%)	22.75	31.75	54.50	
	Yes (%)	22.75	22.75	45.50	χ^2^ = 0.067
	Tot (%)	45.50	54.50	100.00	*p* = 0.560
Hypertension	No (%)	22.75	40.90	63.60	
	Yes (%)	22.75	13.60	36.35	χ^2^ = 1.470
	Tot (%)	45.50	54.50	100.00	p = 0.221
Diabetes mellitus	No (%)	31.80	50.00	81.80	
	Yes (%)	13.70	4.50	18.10	χ^2^ = 1.720
	Tot (%)	45.50	54.50	100.00	*p* = 0.225
Dementia	No (%)	41.00	50.00	91.00	
	Yes (%)	4.50	4.50	9.00	χ^2^ = 0.180
	Tot (%)	45.50	54.50	100.00	*p* = 0.745

*The data are shown as percentages. Among groups, the presence of difference in aspect ratio was analyzed with the Chi square test, statistical significance was attributed as p <0.05*.

**Table 3:** Differences between baseline and follow-up in each group.

	**Control group**			**Treatment group**			**Δ% Baseline—follow-up**		
**Parameters**	**Baseline**	**Follow-up**		**Baseline**	**Follow-up**		**Control group**	**Treatment groups**	
	**Mean ± SD**	**Mean ± SD**	***p***	**Mean ± SD**	**Mean ± SD**	***p***	**Mean ± SD**	**Mean ± SD**	***p***
**NLR**	4.32 ± 3.94	1.80 ± 1.05	0.028*	7.14 ± 8.86	1.61 ± 0.84	0.008*	−1.33 ± 62.31	−32.95 ± 47.74	0.236
**PLR**	94.03 ± 84.58	142.34 ± 61.68	0.110*	305.17 ± 166.07	172.34 ± 76.32	0.003*	41.40 ± 5.77	−33.25 ± 5.56	0.017
**PaO**_**2**_ **(mmHg)**	86.20 ± 17.12	86.00 ± 12.81	0.987	79.25 ± 12.01	94.18 ± 18.04	0.045	3.29 ± 20.89	20.01 ± 29.18	0.186
PaCO_2_ (mmHg)	39.20 ± 4.76	32.00 ± 13.29	0.891*	42.08 ± 11.73	39.18 ± 4.49	0.824*	−1.27 ± 9.94	−3.36 ± 18.34	0.774
**FiO**_**2**_ **(%)**	0.31 ± 0.12	0.23 ± 0.05	0.317*	0.29 ± 0.12	0.22 ± 0.03	0.042*	−7.25 ± 20.51	−16.34 ± 20.27	0.350
**PaO**_**2**_**/FiO**_**2**_	304.35 ± 109.35	351.62 ± 72.05	0.042	315.67 ± 123.06	437.58 ± 99.27	0.014	14.93 ± 16.94	55.77 ± 15.06	0.046
**RBC (10**^**3**^**/μL)**	4.85 ± 0.38	4.57 ± 0.32	0.032	4.43 ± 0.73	4.27 ± 0.77	0.358	−4.18 ± 4.98	−2.98 ± 12.18	0.785
**Hemoglobin (g/dL)**	13.65 ± 1.73	12.98 ± 1.63	0.032	13.23 ± 2.39	12.61 ± 2.07	0.256	−3.44 ± 4.65	−3.54 ± 12.81	0.981
**Platelets (10**^**3**^**/μL)**	147.52 ± 126.17	256.78 ± 85.30	0.021	230.92 ± 122.42	258.58 ± 96.92	0.293	80.02 ± 46.38	23.19 ± 43.44	0.013
WBC (10^3^/μL)	8.78 ± 4.41	6.49 ± 3.32	0.066*	5.58 ± 2.94	5.59 ± 2.34	0.638*	−19.92 ± 31.62	11.84 ± 42.51	0.075
**Neutrophils (10**^**3**^**/μL)**	6.25 ± 4.57	3.57 ± 2.83	0.038*	3.98 ± 3.10	3.25 ± 2.18	0.182*	−31.85 ± 42.79	−2.31 ± 50.76	0.175
**Lymphocytes (10**^**3**^**/μL)**	1.74 ± 0.63	1.93 ± 0.53	0.214*	1.01 ± 0.72	1.58 ± 0.42	0.013*	10.87 ± 8.93	56.63 ± 16.59	0.047
**C-reactive Protein (mg/L)**	59.13 ± 76.79	24.92 ± 1.10	0.068*	49.70 ± 47.71	3.30 ± 0.49	0.005*	-54.99 ± 21.46	-92.62 ± 31.10	0.046
Glycemia (mg/dL)	107.3 ± 13.71	104.5 ± 20.33	0.760*	99.58 ± 14.11	93.94 ± 20.39	0.308*	−1.19 ± 23.10	−3.38 ± 27.28	0.843
Albumin (g/dL)	4.01 ± 0.47	3.65 ± 0.46	0.095	3.82 ± 0.63	3.65 ± 0.53	0.284	−5.33 ± 31.21	1.90 ± 40.60	0.443
Sodium (mEq/L)	135.8 ± 4.89	133.3 ± 2.98	0.135	134.58 ± 4.29	136.50 ± 3.45	0.136	−*8, 00*±14.49	−3.11 ± 14.65	0.033
Potassium (mEq/L)	4.28 ± 0.52	4.14 ± 0.40	0.394	3.80 ± 0.38	4.12 ± 0.37	0.066	−1.75 ± 3.55	1.49 ± 3.09	0.059
AST (U/L)	34.6 ± 27.35	39.90 ± 13.23	0.721*	53.67 ± 43.60	42.67 ± 20.11	0.409*	−2.41 ± 1.57	9.36 ± 5.27	0.094
ALT (U/L)	42.50 ± 31.11	85.40 ± 35.89	0.114*	59.50 ± 56.02	68.75 ± 43.33	0.724*	36.57 ± 13.9	23.19 ± 11.09	0.535
GGT (U/L)	43.50 ± 26.69	47.00 ± 23.69	0.314*	38.17 ± 29.03	56.08 ± 65.81	0.875*	3.89 ± 1.11	19.23 ± 4.19	0.416
Indirect bilirubin (mg/dL)	0.64 ± 0.39	0.50 ± 0.16	0.646*	0.91 ± 0.77	0.65 ± 0.17	0.814*	20.89 ± 42	28.04 ± 4.11	0.655
Direct bilirubin (mg/dL)	0.27 ± 0.09	0.26 ± 0.06	1.000*	0.23 ± 0.14	0.20 ± 0.13	0.625*	−4.35 ± 7.45	5.02 ± 5.48	0.450
**Amylase (U/L)**	87.2 ± 44.34	92.16 ± 30.50	0.097*	76.92 ± 43.84	59.38 ± 16.62	0.025*	6.91 ± 4.44	−23.29 ± 1.47	0.227
**Lipase (U/L)**	178.5 ± 193.15	258.40 ± 160.45	0.037*	143.42 ± 105.84	103.30 ± 36.9	0.042*	20.92 ± 5.89	−5.73 ± 4.33	0.661
Fibrinogen (mg/dL)	554.00 ± 265.23	308.75 ± 62.11	0.144*	459.25 ± 231.85	383.44 ± 226.78	0.345*	−53.99 ± 17.42	−20.37 ± 17.48	0.104
**D-Dimer (ng/mL)**	736.20 ± 564.55	525.57 ± 888.74	1.000*	346.67 ± 309.91	232.33 ± 280.73	0.020*	−28.86 ± 30.33	−8.65 ± 18.71	0.348

Parameters are presented as mean ± standard deviation and Δ% between Baseline and Follow-up. Differences between baseline and follow-up in each group were compared by Dependent samples t-test and (*) Wicoxon test, while Δ% Baseline—Follow-up differences between groups were compared by ANOVA one way test. Statistical significance was attributed as p <0.05. SD, standard deviation; BMI, body mass index; HDCL, hydroxychloroquine; AZT, azithromycin; PaO_2_, arterial oxygen partial pressure; PaCO_2_, arterial carbon dioxide partial pressure; FiO_2_, fractional inspired oxygen; PaO_2_/FiO_2_, arterial oxygen partial pressure and fractional inspired oxygen ratio; NLR, neutrophils and lymphocytes ratio; PLR, platelets and lymphocytes ratio; RBC, red blood Cells; WBC, white blood cells; AST, aspartate aminotransferase; ALT, alanine aminotransferase; GGT, gamma-glutamyl transpeptidase.

**Table 4:** Table contingency of qualitative change after 21 days in finding CT.

	**Overall**				**Control group**				**Treatment group**			
**Parameters**	**Baseline**	**21st day**	**χ^**2**^**	***p***	**Baseline**	**21st day**	**χ^**2**^**	***p***	**Baseline**	**21st day**	**χ^**2**^**	***p***
**Presence of:**												
**Ground-glass opacities or consolidation**	64.29	0.00	9.95	0.002	83.33	0.00	5.33	0.021	50.00	0.00	5.33	0.021
Ground-glass opacities without consolidation	28.57	50.00	1.34	0.246	0.00	33.33	2.40	0.121	50.00	62.50	0.25	0.614
**Ground-glass opacities with consolidation**	50.00	7.14	6.30	0.012	66.67	16.67	3.08	0.079	37.50	0.00	3.70	0.045
Consolidation without ground-glass opacities	21.43	21.43	0.00	1.000	33.33	33.33	0.00	1.000	12.50	12.50	0.00	1.000
**Lobes affected**												
**More than two lobes affected**	42.86	64.29	0.70	0.403	0.00	83.33	5.33	0.035	75.00	50.00	1.07	0.302
**Bilateral lung disease**	42.86	78.57	0.00	1.000	0.00	83.33	5.33	0.035	75.00	75.00	0.00	1.000
**Frequency of lobe involvement**												
Right upper lobe	71.43	57.14	0.62	0.430	83.33	66.67	0.44	0.505	62.50	50.00	0.25	0.614
Right middle lobe	78.57	64.29	0.70	0.403	83.33	83.33	0.00	1.000	75.00	50.00	1.07	0.302
Right Lower lobe	85.71	78.57	0.24	0.622	83.33	83.33	0.00	1.000	87.50	75.00	0.41	0.502
Left upper lobe	85.71	57.14	2.80	0.094	83.33	66.67	0.44	0.505	87.50	50.00	2.67	0.106
Left lower lobe	85.71	71.43	0.84	0.357	100.00	83.33	1.09	0.296	75.00	62.50	2.93	0.590
**Opacification distribution and pattern**												
**Rounded morphology**	71.43	28.57	5.14	0.023	50.00	33.33	0.34	0.558	87.50	25.00	6.34	0.012
Linear opacities	42.86	57.14	1.47	0.225	0.00	66.67	0.44	0.505	75.00	50.00	1.07	0.302
Crazy-paving pattern	21.43	0.00	3.36	0.067	33.33	0.00	2.40	0.121	12.50	0.00	1.07	0.302
Cavitation	7.14	7.14	0.00	1.000	0.00	0.00	//	//	12.50	12.50	0.00	1.000
**Other findings**												
Discrete pulmonary nodules	64.29	28.57	3.60	0.058	83.33	50.00	1.50	0.221	50.00	12.50	2.62	0.106
**Pleural effusion**	64.29	14.29	7.33	0.007	83.33	16.67	5.33	0.021	50.00	12.50	3.29	0.048
**Pneumopathy** **>** **25%**	71.43	35.71	3.56	0.041	83.33	50.00	1.50	0.221	62.50	25.00	3.29	0.048
**Pneumopathy** **>** **50%**	42.86	7.14	4.72	0.029	66.67	16.67	3.08	0.079	25.00	0.00	2.29	0.131
Pulmonary Fibrosis	42.86	35.71	0.15	0.699	83.33	66.67	0.44	0.505	12.50	12.50	0.00	1.000

*The data are shown as percentages. Between time in overall and each time, the presence of qualitative change was analyzed with the Chi square test, statistical significance was attributed as p <0.05*.

## Discussion

The use of inhaled adenosine in COVID19 patients has allowed reduction of LoS, on average 6 days. This result is strengthened by the decrease in SARS-CoV-2 positive days. In TG patients compared to CG, a clear improvement in PaO_2_/FiO_2_, Horowitz index, was observed together with a reduction in inflammation parameters, such as the decrease of CRP level. In addition, an improvement in lymphopenia as a result of reduced inflammation was highlighted. Furthermore, the presumed efficacy of inhaled exogenous adenosine led to an improvement of the prognosis indices, NLR and PLR. This result was confirmed by the reduction in LoS.

In spite of specific antiviral treatment and recent availability of preventative vaccine for COVID-19, large numbers of patients are still being admitted with long hospitalizations and high mortality rate, two of the main drivers of major public health concerns. In COVID-19, lopinavir/ritonavir did not show an improvement in the mortality rate.

Indeed, several treated patients had to discontinue treatment due to gastrointestinal side effects. Also, the randomized study on treatment with TCZ, administered at an early stage, ended prematurely due to the absence of benefit in terms of aggravation and survival (54). Again, on November 20th, the WHO pointed out that there is not enough evidence to support its use. Indeed, regardless of disease severity, remdesivir only has conditional recommendation for use in COVID 19 (55).

Besides, no significant differences in clinical status or overall mortality were observed between patients treated with convalescent plasma and those who received placebo (56).

By harnessing the regulatory effects of adenosine on inflammatory mediators, we have instituted a new therapeutic treatment with inhaled adenosine in COVID-19 patients, with the aim of reducing inflammation, the onset of cytokine storm, and therefore to improve prognosis.

At baseline, patients showed no differences between the two groups in terms of age, BMI, standard therapy duration, and respiratory parameters. Furthermore, no differences were observed in the anamnestic data and in the use of respiratory support. LoS reduction, one of the two primary outcomes, was observed in TG and between groups the difference was approximately of 6 days. This result affects the clinical, economic, and social aspect. In addition, decreased risk of complications, the psychological impact and the consequent recovery after hospitalization are highlighted (57).

Given the emergency, isolated hospitalization, far from relatives and in absence of the usual physician-patient relationship, had a negative impact on psycho-physical health (49, 58). The reduction of positive days to Sars-CoV2 is a significant finding that confirms the above-mentioned results. In fact, TG patients showed a negative detection of SarS-CoV2 approximately 11 days sooner. LoS only included the stay in Infectious Disease Department, while positivity days also included hospitalization in the Emergency Department. In TG, the LOS exceeds the positive period of 1 day, this is due to the waiting of clinical recovery.

At baseline, severe lymphopenia, leukopenia, elevated levels of CRP, D-Dimer, lipase, and amylase were present in both groups, clinical features already observed in other studies (59–62). In TG, lymphopenia was higher in comparison to CG, with a consequent higher PLR. As already observed by Chen et al. (63) and by our group, lymphopenia is a feature present in symptomatic patients and with severe COVID-19. The high production of cytokines and inflammation leads to a decrease in T lymphocytes proliferation and exhaustion of their immunological profile (64). Probably, an unresponsive lymphocyte function is the host countermeasure to prevent mass immune activations and consequent tissue damage. Finally, we hypothesize that some clinical differences at baseline are attributable to the heterogeneity of patients who had access to the GOM in the emergency phase.

As Wang et al. (62) signs of pancreatic distress were observed, a common feature with previous Sars-CoV infection. In fact, the ACE2 is expressed in the intestine and pancreas (65, 66). In addition to the cytopathic effect induced by viral replication, the pancreatic insult could be caused by the action of the immune system in response to lung and systemic inflammation (62).

At follow-up, the results showed a general improvement in clinical conditions, inflammatory and respiratory parameters in both groups, a course linked to the disease (9, 67, 68). In both groups, an improvement in the PaO_2_/FiO_2_ ratio was observed. However, the TG showed greater improvements. At the same time, a reduction of oxygen therapy administered and a significant increase in PaO_2_ was observed in TG. When comparing the groups, Δ% of PaO_2_/FiO_2_ confirmed the effectiveness of the treatment with a significant increase of 55% for TG. The hypothesized beneficial effect of adenosine is observed from the improvement of lung function and from the signs of inflammatory decrease in TG. Alongside, increase in PaO_2_/FiO_2_, a significant reduction in C-reactive protein, a resumption of lymphocyte count and a reduction in D-Dimer levels were observed. Similar results were seen in patients treated with Acalabrutinib, an inhibitor of the Bruton tyrosine kinase enzyme (69).

The adenosine effectiveness could be attributed to the modulating and regulatory activity on macrophages. In fact, signs of macrophage activation syndrome were observed in CG, such as an increase in CRP, inflammatory cytokines (70) and the predominant presence of monocytes/macrophages in the pulmonary alveoli, observed in post-mortem analyses (71).

In patients with COVID-19 obesity and related diseases has been found to be more common, depicting a higher inflammatory “Starting Point” determining activation and dysregulation of macrophages (72, 73). Proper functioning of the innate immune system allows for the rapid recognition and elimination of pathogens (74). In COVID 19, the presence of a dysregulated immune response (17, 67) with a proinflammatory imbalance, an excessive recruitment of monocytes, macrophages and overproduction of cytokines and inflammatory molecules has been observed in some cases (75, 76).

It can be hypothesized that those events determine the pulmonary macrophage activation syndrome and, in some cases, the systemic trigger of the cytokine storm.

In this process, adenosine administered by inhalation helps regulating inflammation through its receptors, A2A and A2B expressed in macrophages, neutrophils, monocytes, lymphocytes and platelets (77–79).

We hypothesized that the treatment is aimed at restoring the signaling mediated by A2A and A2B AR and therefore increasing the protection of healthy lung tissue by increasing the protective capacity of the respiratory epithelial barrier and reducing the damage induced by overactivation of the immune system. From this perspective, there is broad agreement that mechanical lung damage associated with active ventilation may add to SARS-CoV-2-induced lung damage. On the other hand, it is now widely known that the abuse of hyperoxic respiration itself can inhibit the main physiological protective mechanism of hypoxia-A2-adenosinergic which leads to massive consequences of tissue damage (80).

It has been shown that extracellular accumulation of adenosine can contribute to regulating inflammation, immunity, and tissue repair (75).

Through A2A, adenosine determines the differentiation of an anti-inflammatory dendritic phenotype that promotes TH2 lymphocytes, inhibition of T-cell receptor signaling and generation of regulatory TH17 (81). Furthermore, the activation of A2B leads to the inhibition of activating macrophages with inhibition of the release of TNF-α, IL-6 and IL-12, increasing the production of the anti-inflammatory molecule IL-10 (82).

In addition, a significant decrease in D-dimer, an increase in circulating lymphocytes and a decrease in NLR and PLR were observed in TG. This is due to the reduction of inflammation, probably mediated by the treatment. The same improvements are not observed in the CG, but a reduction in the number of neutrophils is highlighted.

However, ours was a case control study and not a randomized double-blind study and lymphocyte count was lower in the treatment group before Adenosine was administered. Therefore, firm conclusions cannot be drawn in comparison with the CG.

Since the NLR index is reduced in both CG and TG, it would not seem to be attributable to the treatment. Indeed, observing the terms of the NLR ratio, the reduction of neutrophils is observed in the CG, while in the TG there is a recovery of lymphopenia. Therefore, the result might seem the same but is linked to different inflammatory processes and to disease course.

Again, the observed reduction in TG of PLR is compatible with an improvement in the prognosis, which is then confirmed by the reduction in LoS (60).

At the follow-up, a worsening of pancreatic indices, in particular lipase was observed in CG. On the other hand, the opposite was observed in TG, with a significant improvement in both pancreatic enzymes. This could be explained by the reduction of lung and systemic inflammation and by pancreatic localization of adenosine receptors (83).

Regarding CT findings, reductions of ground-glass opacities with consolidation and rounded morphology were observed in TG. Then, pleural effusion and pneumopathy under the 50% reduction were observed. The radiological improvement supports the hypothesis of this study since there is a regression of inflammation and lung lesions.

Finally, adenosine use showed a good tolerance of the administered dose with no cardiovascular and respiratory side effects, confirming the safety of its use.

The hypothesis presented by Abouelkhair (84) on the use of drugs inhibiting adenosine formation and its pathways with the increase of ATP levels, does not find a rationale in the timing of administration.

In fact, with current knowledge it is difficult to understand when to administer these drugs. Therefore, compared to the previous hypothesis, the treatment and the route of administration we propose finds a good time of administration: the symptomatic patient and before clinical features of the onset of cytokine storm are seen.

Given the beneficial effects of adenosine in acute inflammation, we propose a new treatment for hospitalized COVID-19 patients, with the aim to prevent the cytokine storm, and therefore to improve the prognosis, and reducing LoS. The study was conducted as case-control but randomized controlled-clinical trials on a larger population will be necessary to confirm our results (85). Limitations of this study include the lack of studies on involved molecular pathways, dosage of inflammation biomarkers, small sample size, and non-randomization, due to emergency and off-label treatment. The case-control study design limits the results interpretation. Thus, there is a definite necessity for a randomized multicenter study, which also involves the use of specific biomarkers useful for describing inflammatory responses.”

Continuous chronic administration of inhaled adenosine has been demonstrated to give rise to the onset of fibrosis and pulmonary hypertension (32). These could be potential negative consequences, especially after a Sars-CoV-2 infection. Consequently, adenosine treatment was limited to 5 days to avoid development of fibrosis. Moreover, long-term monitoring was provided for these patients.

Future research should also consider the evaluation of adenosine in ARDS patients. Also, it would be useful to introduce preclinical studies that take into consideration the entire immune profile for a full comprehension of underlying mechanisms, potentiality, and limits. Supportive evidence in theory has been published in two key papers. Oral Pentoxyfylline has a similar effect to inhaled adenosine in working *via* the A2 AR pathway to attenuate immune exuberance by increasing the secondary messenger Cyclic AMP in the A2 AR pathway beyond adenosine (86, 87).

In conclusion, the treatment seems to be safe and modulates the immune system, allowing an effective response against the progress of the viral infection, reducing LoS. In fact, a reduction in inflammation, resumption of immune response parameters, prognostic indices, clinical pulmonary and systemic improvement are reported. Furthermore, the hypothesis that the treatment leads to an improvement in the respiratory parameters *a priori* in COVID-19, due to a direct action on the respiratory tract (88), remains to be investigated.

## Data Availability Statement

The raw data supporting the conclusions of this article will be made available by the authors, without undue reservation.

## Ethics Statement

The studies involving human participants were reviewed and approved by Ethics Committee of Southern Calabria (April 30, 2020). The patients/participants provided their written informed consent to participate in this study.

## Author Contributions

MCc, PC, and AD: conceptualization. LR: methodology. LDR and LR: formal analysis. MCc, PC, CM, GF, and SM: investigation. CF, MCu, MCo, ACF, EI, AM, MCg, NP, AF, DL, and MT: data curation. LDR, LR, and AD: writing—original draft preparation. LDR, GC, LR, and AD: writing—review and editing. LDR and LR: visualization. AD: supervision. All authors have read and agreed to the published version of the manuscript. Authorship must be limited to those who have contributed substantially to the work reported.

## Conflict of Interest

The authors declare that the research was conducted in the absence of any commercial or financial relationships that could be construed as a potential conflict of interest.

## Abbreviations

A2AR: adenosine 2A receptors
A2BR: adenosine 2B receptors
ACE: angiotensin converting enzyme 2
ALT: alanine aminotransferase
AR: adenosine receptors
ARDS: acute respiratory distress syndrome
AST: aspartate aminotransferase
B: B lymphocyte
BMI: body mass index
cAMP: cyclic AMP
CG: control group
CKF: chronic kidney failure
COPD: chronic obstructive pulmonary disease
COVID-19: coronavirus disease 2019
CREB: reactive element of cAMP
CRP: C-reactive protein
CT: computer tomography
CTL: cytotoxic
FiO_2_: inspired oxygen fraction
GGT: gamma-glutamyl transpeptidase
GOM: grande ospedale metropolitano
HCQ: hydroxychloroquine
ICU: intensive care unit
IFN-α: interferon-α
Ig: immunoglobulin g
IL: interleukin
INF-γ: interferon-gamma
LDH: lactate dehydrogenase
LoS: length of stay
LPS: Lipopolysaccharide
mAb: monoclonal antibodies
MCP-1: monocyte chemoattractant protein-1
MERS-CoV: Middle East respiratory syndrome-related coronavirus
MERS-CoV: middle east respiratory syndrome coronavirus
MHC: major histocompatibility class
MI: myocardial infarction
NFκB: nuclear factor kappa-light-chain-enhancer of activated B cells
NK: natural killer cell
NLR: neutrophils to lymphocytes
PaCO_2_: arterial carbon dioxide partial pressure
PaO_2_: arterial oxygen partial pressure
PaO_2_/FiO_2_: arterial oxygen partial pressure and fractional inspired oxygen ratio
PLR: platelet to lymphocyte
RBC: red blood cell
RdRp: RNA-dependent RNA polymerase
SARS-CoV-2: severe acute respiratory syndrome coronavirus 2
T: T lymphocyte
TCR: T-cell receptor-mediated signaling
TCZ: Tocilizumab
TG: treated group
Th: T helper
TLR: toll-like receptor
TNF: tumor necrosis factor
TNF-α: tumor necrosis factor alpha
WBC: white blood cell.
